# Loss of ACSM3 confers worsened prognosis and immune exclusion to cutaneous melanoma

**DOI:** 10.7150/jca.48354

**Published:** 2020-09-23

**Authors:** Zhidong Zhu, Duoqin Wang, Yanyun Shen

**Affiliations:** 1 Department of Cardiology, Huashan Hospital, Fudan University, PR, China; 2 Department of Dermatology, Huashan Hospital, Fudan University, PR, China

**Keywords:** Malignant melanoma, ACSM3, Cancer immunity

## Abstract

**Aim:** Malignant melanoma (MM) is a highly aggressive cutaneous cancer with undetermined underlying genetic disposition. We aim to evaluate prognostic and mechanistic role of ACSM3 in MM.

**Methods:** In silico reproduction of TCGA MM dataset, GEO dataset, GDSC dataset and human protein atlas was performed to establish differential expression of ACSM3. In vitro and in vivo validation using A375 and SKMEL1 MM cells were performed to profile tumorigenic role and functional attribution of the gene.

**Results:** ACSM3 expression was significantly downregulated in MM. Lower expression of ACSM3 conferred worsened prognosis of MM. Lower ACSM3 was observed in Asian ethnicity. Knock-down (KD) and overexpression (OE) of ACSM3 resulted in significant increased and decreased proliferation, invasion and colony formation in MM cells, respectively. Pathway annotation revealed significantly active immune response invoked by ACSM3. Lower ACSM3 expression was associated with decreased CD8+, macrophage and dendritic cell infiltration. Cox regression revealed loss of survival contribution of ACSM3 in the presence of immune infiltrates supporting immune regulatory role of ACSM3. Drug sensitivity analysis revealed BRAF inhibitor PLX-4720 was sensitive in both MM cells. ACSM3 expression showed no correlation with immune checkpoint molecules. Combined ACSM3-OE and PLX-4720 in MM cells showed synergistic inhibition in MM cells and xenograft murine models with no significant toxicity.

**Conclusion:** Loss of ACSM3 was associated with poor prognosis in MM. Overexpression of ACSM3 synergistically inhibited MM with PLX-4720. ACSM3 was potentially associated with immune exclusion in MM. Further validation was warranted in future studies.

## Introduction

Malignant melanoma (MM) is a tumor that occurs in melanocytes of the skin or other organs with underlying genetic and environmental factors 1. The clinical manifestations include tenderness, itching, bleeding, ulcers, etc. 2. The occurrence peaks at 45 to 60 years of age and is more frequent in men. Though cutaneous MM seldom affect Asians, it poses a major health problem worldwide due to its high aggressiveness and unpredictable metastatic potential 3.

Comprehensive understanding of genetic and genomic alteration in MM offers insightful evidence of the driver events in MM and druggable targets not known before the era of next generation sequencing and omics study. The most renowned studies that profile genomic atlas of MM was, but not limited to the TCGA project 4. Later, the TracerX melanoma project seeks to address the immunological landscapes of MM 5, 6. Un like TCGA, the TracerX project focuses on evolutionary trend within MM, including the determination of spatial and temporal changes in immunological, genomic and transcriptomic landscapes, identification of novel molecular drivers, immunotherapeutic targets and assessment of the impact of cytotoxic, immune-modulatory and targeted therapies on both the tumor microenvironment and peripheral blood.

Therefore, integrating multiple omic platforms may identify therapeutic targets with both cancer-intrinsic and immunity-related functions. With this aim, we seek to identify target gene that is immune-related and prognosis-associated by integrating multiple datasets. In silico findings are further validated with in vitro and in vivo assays.

## Materials and Methods

### In silico analysis

We queried mRNA expression of genes of interest in the TCGA SMCM dataset that consisted of 287 cases. The TCGA database was reproduced with the cBioPortal online software via http://www.cbioportal.org/ 7, 8, in which the OncoPrint tab was used to plot genetic alterations of ACSM3. Clinicopathological parameters of MM was also retrieved form cBioPotal for further correlation analysis. We used another online software, TIMER, to analyze gene expression correlations (using partial correlations) and for graph production 9. The UALCAN online analytical software (http://ualcan.path.uab.edu/index.html) was used for differential expression analysis 10. Gene enrichment analysis was first analyzed using the NETwork-based Gene Enrichment (http://net-ge.biocomp.unibo.it/enrich) and then validated using the GSEA approach 11. The GEO dataset was also used to profile differential expression of ACSM3 (https://www.ncbi.nlm.nih.gov/gds). Differential expression at protein level was further validated in the human protein atlas platform (https://www.proteinatlas.org/) by semi-quantitatively reviewing immunohistochemical (IHC) staining.

### Cell lines

Both A375 and SKMEL1 cells were purchased from the cell bank of Chinese Academy of Science and cultured in RPMI-1640 medium. We used TRC to construct shRNA targeting ACSM3 (TRC, http://www.broadinstitute.org/rnai/public/). Two transcripts were selected for testing (TRCN0000434770 and TRCN0000419964). Vectors with resistance to puromycin were constructed and transfected via non-lipofectamine Fugene transfection. After incubation, positive clones were selected by puromycin supplement and control vectors were generated with similar approach.

### Crystal violet assay

Cell proliferation was measured using the crystal violet assay. Cells were seeded at the density of 2500 cells/well and were subsequently treated with crystal violet. 10% of formalin were used for cell fixation after removal of medium. Methanol was used and plates were read at absorbance at 540 nm.

### mRNA expression

Total RNA was extracted with Trizol reagent and was converted to cDNA. We used Trizol for RNA extraction and primer for ACSM3 was constructed with sequence as follows: forward, AGG AAG ATG CTA CGT CAT GCC; reverse, ATC CCC AGT TTG AAG TCC TGT. The Real-time quantitative PCR was performed to determine relative expression level of the genes. For western blotting, total protein was extracted then separated by SDS-PAGE. Proteins were then transferred to a a polyvinylidene difluoride membrane blocked by non-fat milk.

### Flow cytometry

The FASCanto flow cytometry system was used to measure cell cycle and apoptotic profiles.

For cell cycle analysis, cells were fixed using cold ethanol and later treated with cell cycle staining buffer. For apoptosis, cells were applied with Annexin V and PI and apoptotic cells were defined as sum of early and late apoptotic cells.

### Transwell assays

Both invasion and migration were measured by Transwell assay. Cells were seeded in the upper chamber of the Transwell plate at the density of 1×10^6^/ml, either coated (for invasion) and uncoated (for migration) with Matrigel. Upper chamber was supplemented with serum-free media whilst the lower chamber was filled with complete medium. Cells that penetrated were stained with crystal violet and counted for number.

### Colony formation assays

Anchorage-independent growth was measured using colony-formation assay. Cells were seeded in mixture of medium with 0.4% agar, layered on top of 0.6% agar mixed with complete medium. Cells were incubated for 2 weeks and were stained with 0.005% of crystal violet and colonies were counted microscopically.

### Xenograft mouse model

Subcutaneous tumor implantation mouse model was used to profile therapeutic effect in vivo.

Both A375 and SKMEL1 cells were injected s.c. at axillary region of mouse and PLX-4720 was administrated through ad libitum chow at a dose of 417 mg/kg, whilst LvACSM3 and control plasmid was overexpression was delivered to both cell lines before implantation. Mice were euthanized on Day 35 and tumor size was calibrated using the formula Length * Width^2^ * 0.5236. All animal experiments were approved by the Department of Lab Animal Science of Fudan University.

### Statistical analysis

Statistical analysis for in silico studies were automatically performed with the platforms used, as aforementioned. Statistical analysis for in vitro assays were performed using the Prism Graphpad 7.0 for Mac. Comparisons between two groups were studied using the Student's t test. The P value of < .05 was accepted as significant.

## Results

### Lower expression of ACSM3 confers worsened prognosis in MM

**Fig 1:**
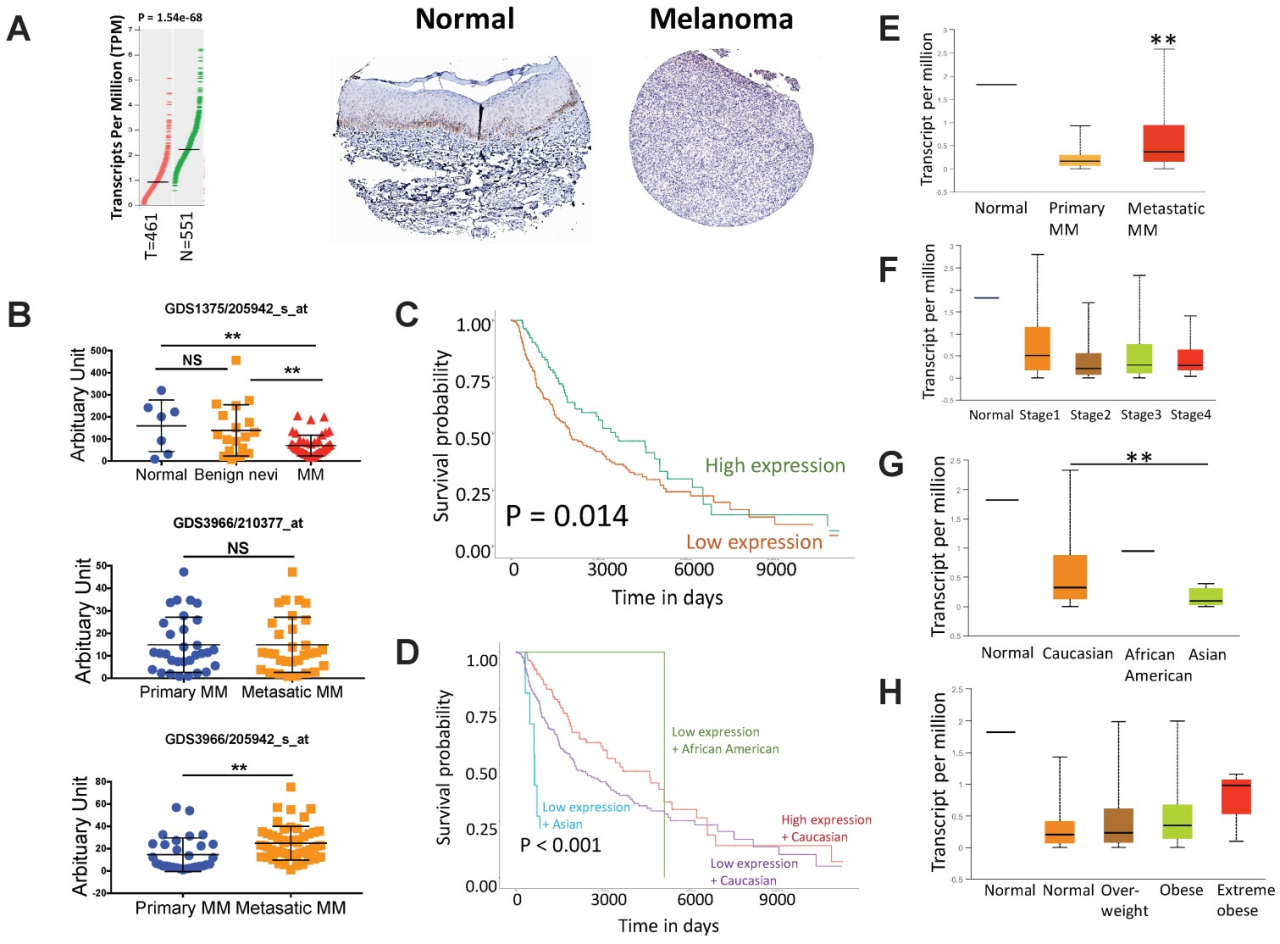
ACSM3 loss is associated with progression of malignant melanoma (MM). A) reproduced from TCGA dataset using GEPIA, shown was differential expression of ACSM3 between normal (N) and tumor (T) tissue with representative immunohistochemical images of ACSM3; B) Reproduction of GEO dataset showing differential expression of ACSM3 between normal, benign nevi and MM tissue; C) Overall survival between patients with high and low ACSM3 expression; Subgroup analysis of D) survival between ACSM3 expression and different races, E) ACSM3 expression in primary and metastatic diseases, F) ACSM3 expression in different stages, G) ACSM3 expression in different races and H) ACSM3 expression in different weight scales.

We first evaluated expression of ACSM3 in MM tissue and normal skin tissue by reproducing TCGA and HPA datasets. We found ACSM3 was differentially expressed in MM, showing significant loss in cancer tissue (**Fig. 1A**). Further validation in 3 GEO datasets confirmed our findings showing significant lower ACSM3 expression in MM whilst unchanged ACSM3 level between benign nevi and normal skin (**Fig. 1B**). GEO also demonstrated inconsistent ACSM3 expression in metastatic MM compared with primary entity (**Fig. 1B**). Lower ACSM3 expression conferred significantly worsened prognosis in MM and survival was poorest in Asian MM patients (**Fig. 1C-D**). Subgroup analysis showed significantly higher ACSM3 expression in metastatic MM (**Fig. 1E**). However, ACSM3 was not differentially expressed across stages (**Fig. 1F**). Expression of ACSM3 was lowest in Asian MM cases (**Fig. 1G**). A trend towards higher ACSM3 expression in obese patients was noticed (**Fig. 1H**).

### ACSM3 overexpression inhibits MM growth

**Fig 2:**
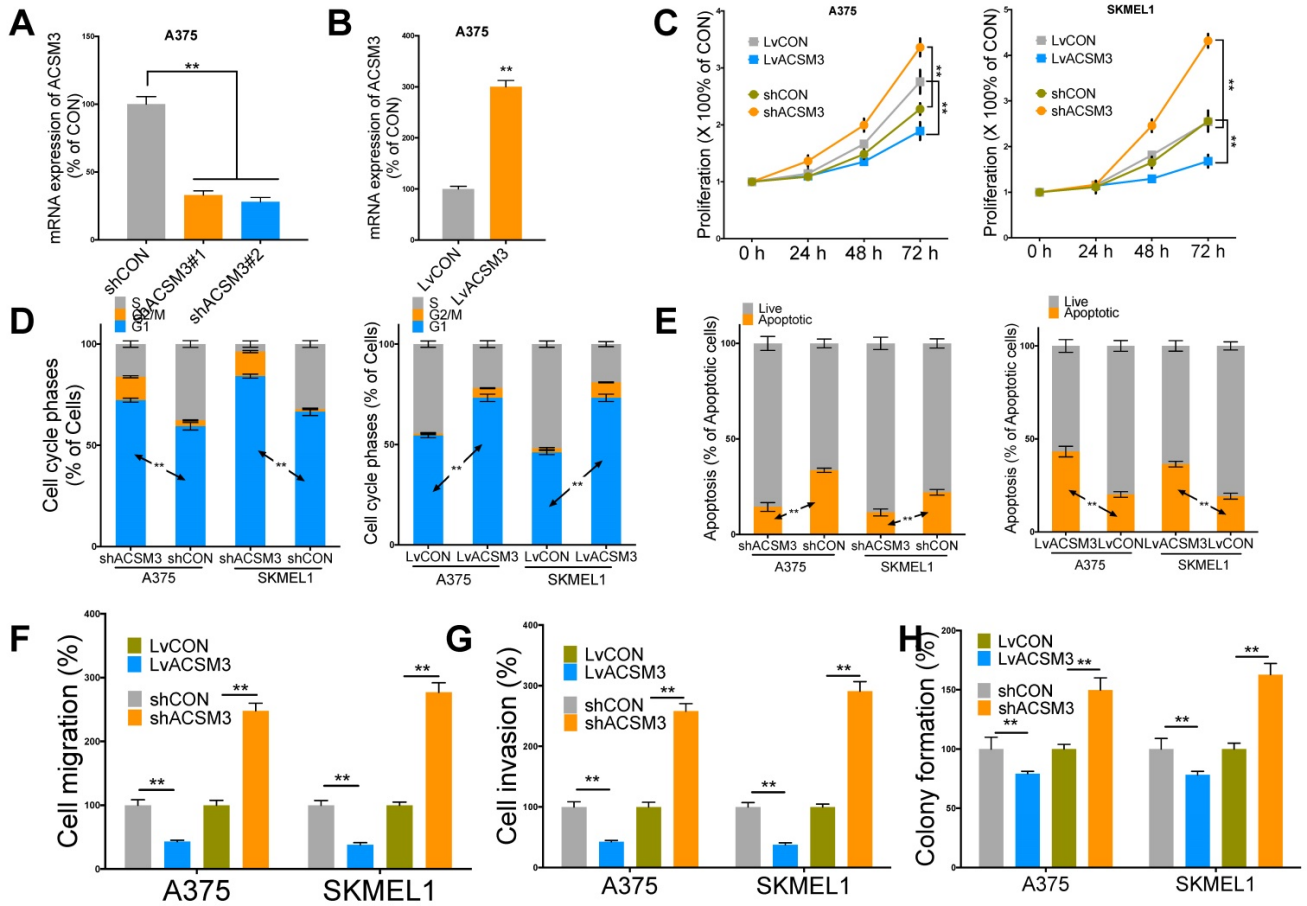
ACSM3 inhibits malignant melanoma (MM) growth in vitro. A) Efficiency of knockdown (KD) two transcripts of shRNA targeting ACSM3 selected on TRC demonstrated by quantitative PCR, B) Efficiency of overexpression (OE) of ACSM3 delivered by cDNA-bearing lentivirus; C) Proliferation of both MM cells interfered by shRNA (#2) and lentivirus detected by crystal violet assay at each time point given; Flow cytometry detecting D) cell cycle profile of MM cells interfered by ACSM3 OE and KD using Propidium Iodide (PI) staining and detecting E) cell apoptosis using PI and Annexin V staining; Transwell assays detected at 72 h of viral infection of both OE and KD of ACSM3 in MM cells showing effect in F) migration and G) invasion; H) colony formation detected at week 2 of MM cells with ACSM3 OE and KD using agarose assays (*P < 0.05; **P < 0.01).

We then performed in vitro validation of the findings in silico. We performed effective knockdown (KD) and overexpression (OE) of ACSM3 in MM cell lines (**Fig. 2A-B**). ACSM3-KD significantly increased proliferation and ACSM3-OE significantly decreased proliferation of both MM cells (**Fig. 2C**). ACSM3-KD significantly decreased population in G1 phase and ACSM3-OE significantly increased population in G1 phase in both MM cells (**Fig. 2D**). ACSM3-KD significantly decreased apoptosis and ACSM3-OE significantly increased apoptosis in both MM cells (**Fig. 2E**). Similar effects were also observed in cell migration, invasion, and colony formation in both cells (**Fig 2F-H**).

### ACSM3 is associated with cancer immunity in MM

**Fig 3:**
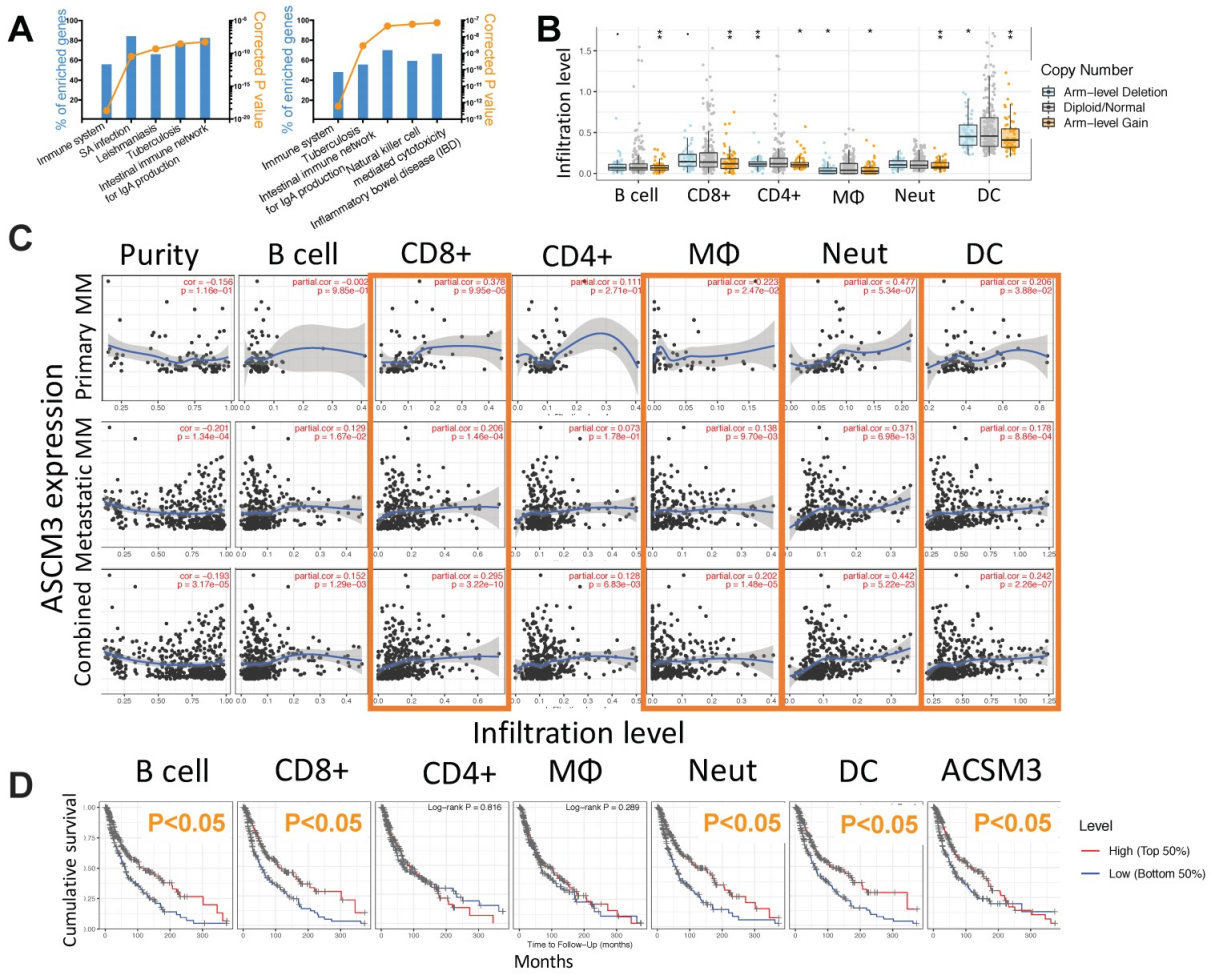
ACSM3 is associated with cancer immunity in MM. Shown were A) NET-GE-based pathway analysis of genes expressed in significant correlation with ACSM3 in MM showing top 5 enriched pathways using standard (left) and network-based (right) analysis; B) relation between immune infiltrates and copy number of ACSM3 in MM; C) Partial correlation analysis curve fits between ACSM3 expression and different tumor infiltrating lymphocytes (TIL); D) Overall survival of MM patients with higher 50% or lower 50% of TIL infiltration (*P < 0.05; **P < 0.01).

**Fig 4:**
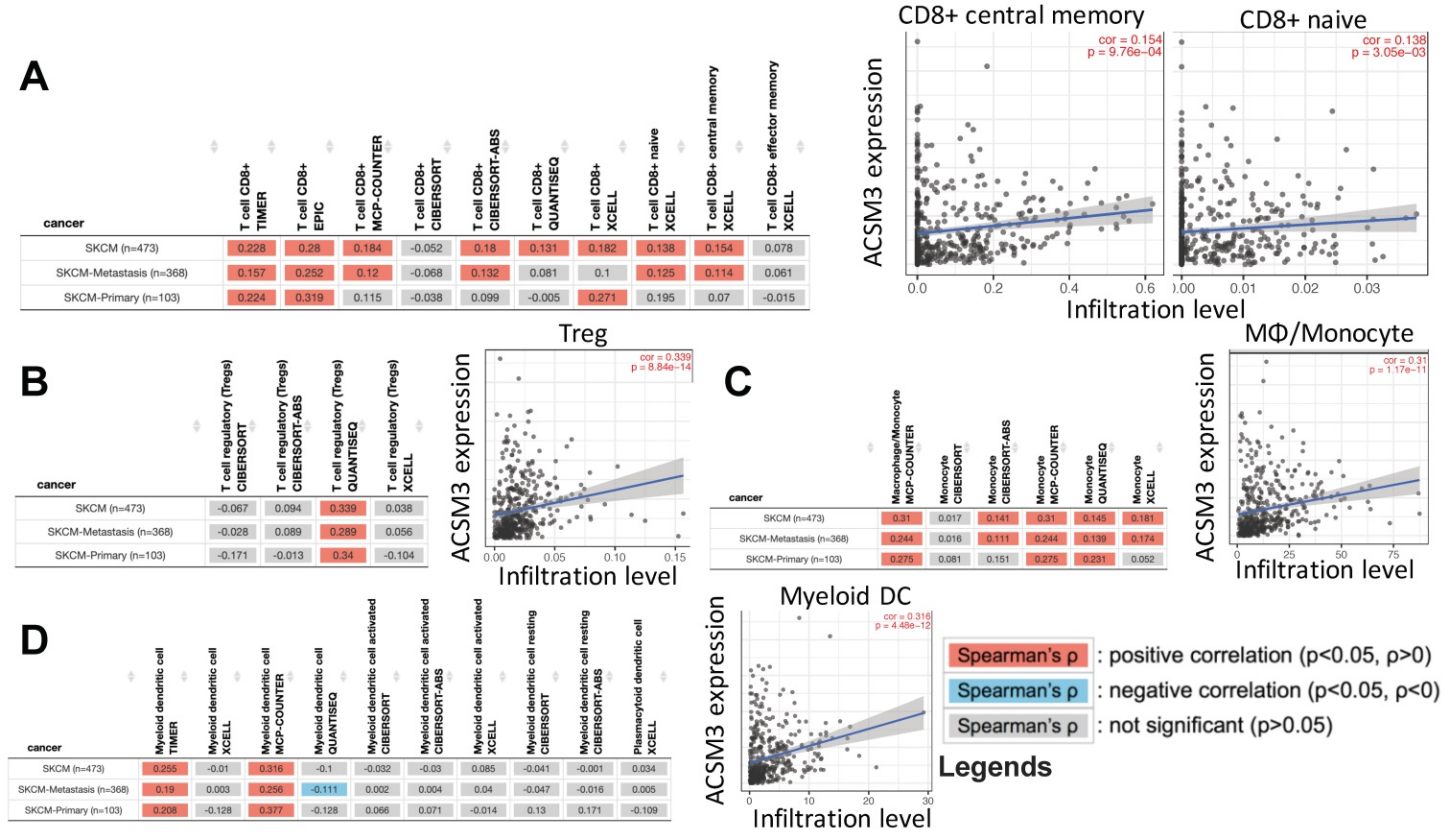
ACSM3 expression was associated with infiltration of certain TIL types in MM. reproduced from TCGA dataset using TIMER 2.0, shown were correlations between ACSM3 expression and A) CD8+ subtype, B) regulatory T cells (Treg), C) macrophage/monocyte and D) myeloid dendritic cells with different calculation algorisms.

To profile mechanistic attribution of ACSM3 we performed enrichment analysis for genes expressed in significant correlation with ACSM3 in MM and showed significant enriched genes within immune-regulatory pathways by both standard enrichment analysis (**Fig. 3A left**) and network-based enrichment analysis (**Fig. 3A right**). As ACSM3 presented heterozygous loss in MM, we plotted ACSM3 copy number against immune infiltrates level and found Higher ACSM3 copy number was associated with less CD8+ T cells, neutrophils and DCs (**Fig. 3B**). We then performed breakdown analysis of ACSM3 expression and distinct TIL types. We highlighted consistent and significant correlations in both primary and metastatic MM, and found ACSM3 expression was associated with infiltration of CD8+ T cells, macrophage, neutrophils, and DCs (**Fig 3C**). We then investigated role of immune infiltrates on survival and found that lower B cell, CD8+ cell, neutrophil and DC infiltration was associated with decreased survival, respectively (**Fig. 3D**). Taken together, we performed Cox regression with all elements passing significance included in the multivariate analysis and found race, tumor stage, DC being independent prognostic factors, whist ACSM3 remaining marginal significance (**Table 1**). We further studied correlation between ACSM3 expression and immune infiltrate subtypes and found that ACSM3 was positively correlated with CD8+ central memory and naïve cells (**Fig. 4A**), T regulatory cells (Tregs) (**Fig. 4B**), macrophages (**Fig. 4C**) and DCs (**Fig. 4D**).

### Overexpression of ACSM3 synergistically enhances BRAF inhibition in MM

**Fig 5:**
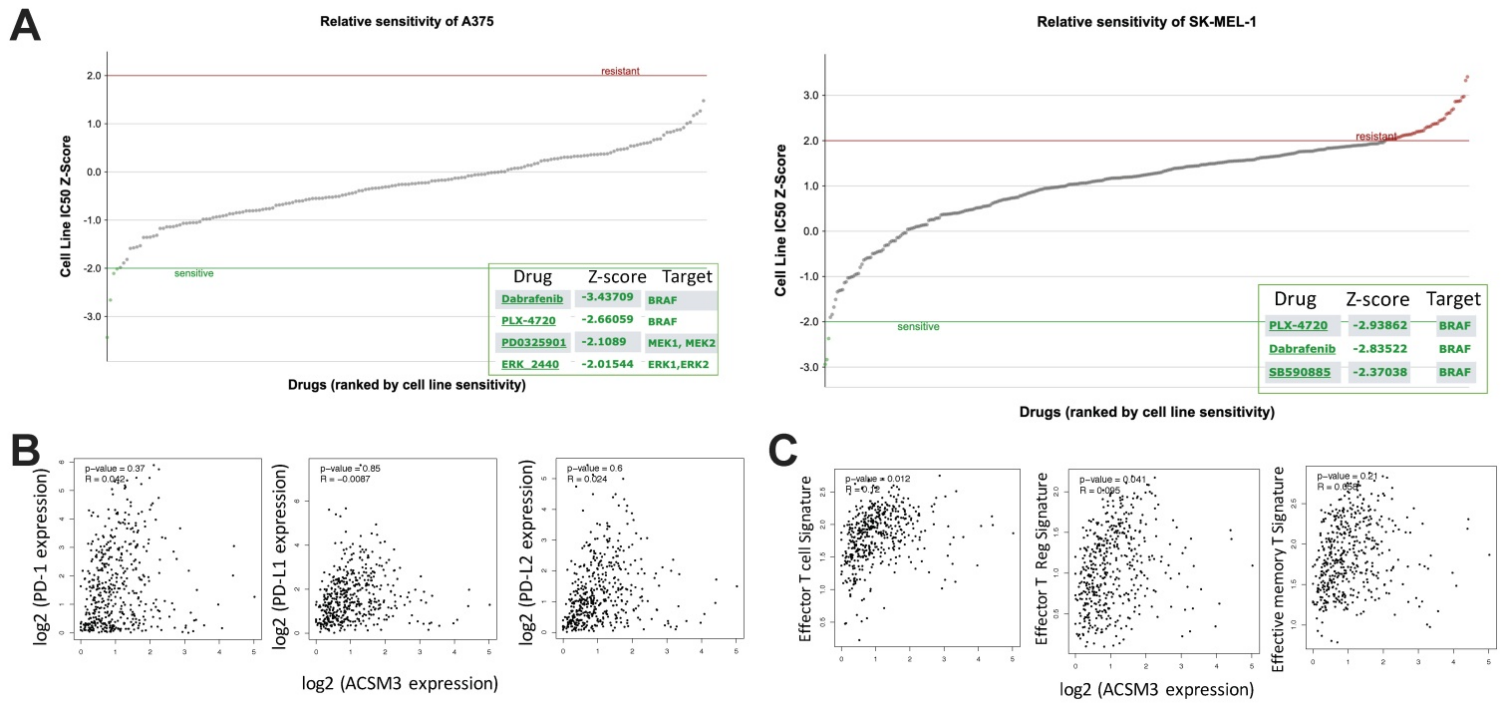
In silico exploration of potential inhibitory compounds in malignant melanoma (MM) cells. A) Drug sensitivity analysis reproduced from GDSC dataset showing significantly sensitive compounds in both MM cell lines; Expression correlation analysis reproduced from TCGA dataset using GEPIA platform showing B) correlations between expressions of immune checkpoint markers and ACSM3, and C) correlations between expressions of T cell signatures and ACSM3.

MM was characterized with activated ERK signaling and BRAF inhibitors have been implicated clinically as one of the major medicinal treatments. We first found both MM cell liens were sensitive to BRAF inhibitor PLX-4720 (**Fig. 5A**). As ASCM3 was associated with cancer immunity, we then examined relations between expressions of ACSM3 and immune checkpoints. We found expression of ACSM3 was not correlated with that of PD-1, PD-L1 or PD-L2 (**Fig. 5B**). Whilst ACSM3 expression was correlated with signatures of effector T cell and Tregs, it was not associated with memory T cell signature (**Fig. 5C**). We therefore further tested combination therapy of ACSM3-OE and PLX-4720.

**Fig 6:**
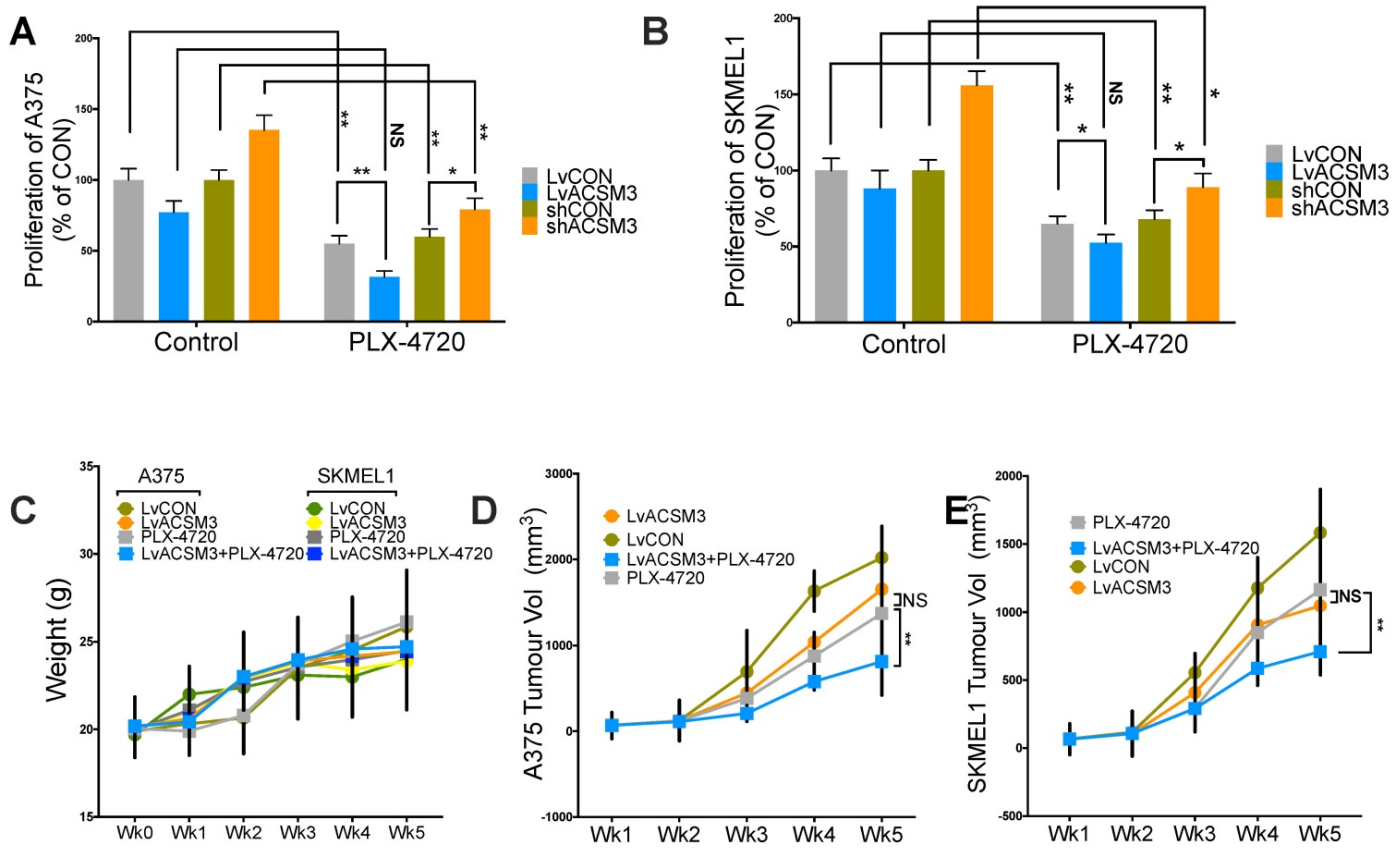
Combination of ACSM3-OE and BRAF inhibition shows synergy in MM. Proliferation assay using crystal violet detected at 72 h of culture showing effect of combination treatment of PLX-4720 at the single dose of 50 nM added in A) A375 and B) SKMEL1 MM cells; MM xenograft mouse model of 6 BALB/c male nude mice of 6 weeks of age per group with combination of PLX-4720 (ad libitum; 417 mg/kg) showing E) weight monitoring of all treatment groups; F) tumor size monitoring between treatment groups of both cell lines (NS, not significant; *P < 0.05; **P < 0.01).

We found combination therapy resulted in synergy in comparison with either monotherapy using proliferation assay (**Fig. 6A**). We then studied xenograft MM murine models and found that no significant toxicity observed across treatment and control groups (**Fig. 6B**). Similar to in vitro findings, MM tumors showed significantly decreased growth in the presence of combination therapy (**Fig. 6C**).

## Discussion

In the current study, we have shown that ACSM3 expression was significantly downregulated in MM. Lower expression of ACSM3 conferred worsened prognosis of MM. Lower ACSM3 was observed in Asian ethnicity. Knock-down (KD) and overexpression (OE) of ACSM3 resulted in significant increased and decreased proliferation, invasion and colony formation in MM cells, respectively. Pathway annotation revealed significantly active immune response invoked by ACSM3. Lower ACSM3 expression was associated with decreased CD8+, macrophage and dendritic cell infiltration. Cox regression reveal loss of survival contribution of ACSM3 in the presence of immune infiltrates supporting immune regulatory role of ACSM3. Drug sensitivity analysis revealed BRAF inhibitor PLX-4720 was sensitive in both MM cells. ACSM3 expression showed no correlation with immune checkpoint molecules. We thus tested combined ACSM3-OE and PLX-4720 in MM cells and found synergistic inhibition in MM cells and xenograft murine models with no significant toxicity.

Cancer immunity is comprised of two aspects, namely immune infiltrate exclusion and negative immune checkpoint upregulation. We speculate that ACSM3 exert the former function in MM. Although we did not test the efficacy of combination of immune checkpoint inhibition (ICI) and ASCM3 OE, we are currently modeling combination of ACSM3 inhibition and ICI as proof-of-concept.

Currently there has been a dearth of studies focusing on ACSM3 in cancer. Two groups reported similar cancer-protective role of ACSM in hepatocellular carcinoma (HCC). Gopal et al used integrative transcriptome analysis of liver cancer profiles and identifies upstream regulators and clinical significance of ACSM3 gene expression 12. They also proposed that differential expression and regulation of the ACSM3 gene in HCC may lay a foundation for therapeutically targeting fatty acid metabolism in these tumors. Likewise, Ruan et al reported downregulation of ACSM3 promotes metastasis and predicts poor prognosis in hepatocellular carcinoma 13. They proposed ACSM3 as a novel prognostic marker and a potential therapeutic target for HCC. Our study echoes their results that ASCM3 loss promotes aggressiveness of MM, and in addition, facilitates immune infiltrate exclusion. Cancer-intrinsic immune exclusion has been addressed in recent year especially in cancers responsible to ICI.

In MM, genes attributive to cancer-intrinsic immune exclusion has been studied as hotspot. Spranger et al reported that the mechanism by which tumor-intrinsic active β-catenin signaling results in T-cell exclusion and resistance to anti-PD-L1/anti-CTLA-4 monoclonal antibody therapy. They propose that specific oncogenic signals, therefore, can mediate cancer immune evasion and resistance to immunotherapies, pointing to new candidate targets for immune potentiation 14. Later on, the β-catenin-driven cancer-intrinsic immune exclusion has been noticed across human cancers. Luke et al reported that using TCGA, a T-cell-inflamed gene expression signature segregated samples within tumor types and found that activation of tumor-intrinsic WNT/β-catenin signaling is enriched in non-T-cell-inflamed tumors. In comparison, the gene we found in this study is no related to β-catenin, but to fat acid metabolism, which has also been reported to exert immune exclusion. Jiang et al reported that ovarian cancer-intrinsic fatty acid synthase prevents anti-tumor immunity by disrupting tumor-infiltrating dendritic cells^15^. Like in our study, we also noticed significantly less DC infiltration in cases with lower ACSM3 expression.

To sum up, we here report loss of ACSM3 was associated with poor prognosis in MM. Overexpression of ACSM3 synergistically inhibited MM. ACSM3 showed possible associated with immune exclusion in MM. ACSM3 hold promise as a novel tumor marker and therapeutic target. Further validation is warranted in future studies.

## Figures and Tables

**Fig 7:**
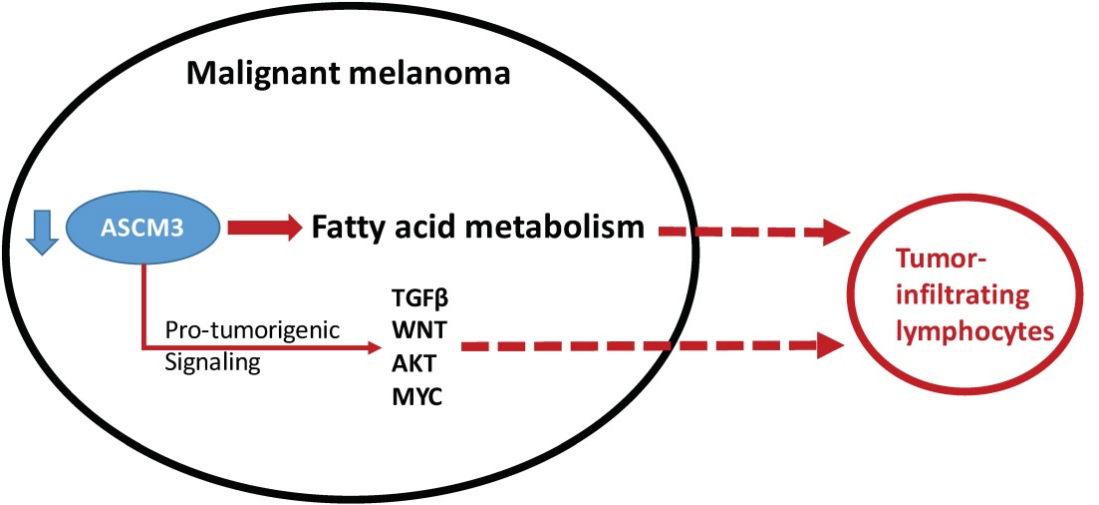
Schematic chart showing functional decreased ACSM3 with reported (solid line) and speculated (dash line) association win pro-tumorigenic signaling and with infiltration of lymphocytes.

**Table 1:** Multivariate analysis of prognostic role of ACSM3 reproduced from the TCGA-SKCM (skin cutaneous malignant melanoma) dataset (430 patients with 211 dying).

	Coefficient	HR	95% Confidence interval		P value
			Lower	Upper	
**Race (Caucasian)**	-1.13	0.323	0.143	0.731	0.007
**Stage 2**	0.379	1.461	0.959	2.225	0.078
**Stage 3**	0.596	1.816	1.206	2.735	0.004
**Stage 4**	1.492	4.444	2.194	9.003	< 0.001
**B cell**	1	2.719	0.121	61.241	0.529
**CD8+ T cell**	0.843	2.323	0.235	22.995	0.471
**Neutrophil**	-2.64	0.071	0	22.002	0.367
**Dendritic cell**	-2.495	0.083	0.012	0.571	0.011
**ACSM3**	-0.191	0.826	0.668	1.021	0.077
